# General Practitioners' Choices and Their Determinants When Starting Treatment for Major Depression: A Cross Sectional, Randomized Case-Vignette Survey

**DOI:** 10.1371/journal.pone.0052429

**Published:** 2012-12-18

**Authors:** Hélène Dumesnil, Sébastien Cortaredona, Hélène Verdoux, Rémy Sebbah, Alain Paraponaris, Pierre Verger

**Affiliations:** 1 UMR912, Sciences Economiques & Sociales de la Santé & Traitement de l'Information Médicale (SESSTIM), Institut National de la Santé et de la Recherche Médicale (INSERM), Marseille, France; 2 UMR-S912, Aix Marseille Université, Institut Recherche et Développement (IRD), Marseille, France; 3 Observatoire Régional de la Santé Provence-Alpes-Côte d'Azur (ORS Paca), Marseille, France; 4 U657, Université Bordeaux, Bordeaux, France; 5 U657, INSERM, Bordeaux, France; 6 Union régionale des professionnels de santé - Médecins libéraux - Provence-Alpes-Côte d'Azur, Marseille, France; The University of Queensland, Australia

## Abstract

**Background:**

In developed countries, primary care physicians manage most patients with depression. Relatively few studies allow a comprehensive assessment of the decisions these doctors make in these cases and the factors associated with these decisions. We studied how general practitioners (GPs) manage the acute phase of a new episode of non-comorbid major depression (MD) and the factors associated with their decisions.

**Methodology/Principal Findings:**

In this cross-sectional telephone survey, professional investigators interviewed an existing panel of randomly selected GPs (1249/1431, response rate: 87.3%). We used case-vignettes about new MD episodes in 8 versions differing by patient gender and socioeconomic status (blue/white collar) and disease intensity (mild/severe). GPs were randomized to receive one of these 8 versions. Overall, 82.6% chose pharmacotherapy; among them GPs chose either an antidepressant (79.8%) or an anxiolytic/hypnotic alone (18.5%). They rarely recommended referral for psychotherapy alone, regardless of severity, but 38.2% chose it in combination with pharmacotherapy. Antidepressant prescription was associated with severity of depression (OR = 1.74; 95%CI = 1.33–2.27), patient gender (female, OR = 0.75; 95%CI = 0.58–0.98), GP personal characteristics (e.g. history of antidepressant treatment: OR = 2.31; 95%CI = 1.41–3.81) and GP belief that antidepressants are overprescribed in France (OR = 0.63; 95%CI = 0.48–0.82). The combination of antidepressants and psychotherapy was associated with severity of depression (OR = 1.82; 95%CI = 1.31–2.52), patient's white-collar status (OR = 1.58; 95%CI = 1.14–2.18), and GPs' dissatisfaction with cooperation with mental health specialists (OR = 0.63; 95%CI = 0.45–0.89). These choices were not associated with either GPs' professional characteristics or psychiatrist density in the GP's practice areas.

**Conclusions/Significance:**

GPs' choices for treating severe MD complied with clinical guidelines better than those for mild MD; GPs rarely recommended psychotherapy alone but rather as a complement to pharmacotherapy. Their decisions were mainly influenced by personal life experience and attitudes regarding treatment more than by professional characteristics. These results call into question the methods and content of continuing medical education in France about MD management.

## Introduction

In developed countries, primary care practitioners manage most cases of depression [1]–[4]. Most research into this management has focused on general practitioners' (GPs) ability to recognize major depression (MD) and their pharmacological treatment of it [5], [6]. Results show that they frequently underdiagnose and undertreat MD. Moreover, at the same time, numerous false positive diagnoses lead to inappropriate antidepressant prescriptions [7]. However, relatively few studies have assessed GPs' decisions about the treatment options for managing MD or the factors associated with these decisions [5], [6], [8], [9]. Yet both French and international guidelines recommend quite different strategies for MD management according to its intensity: in particular, they recommend psychotherapy as an initial treatment of mild to moderate MD and its combination with antidepressants in moderate to severe cases [2], [10]–[12].

Studies that have analyzed the factors associated with GPs' decisions in managing MD have encountered difficulties in distinguishing the factors associated with decisions to treat individuals diagnosed with depression from those associated with the detection of these disorders [8]. Most existing information is also limited by the inability to control for patient characteristics [13]. Finally, studies of GP attendees may suffer from important selection biases due to the complexity of their implementation and thus the likelihood that both GPs and patients will perceive them as intrusive [5], [14]. Selection biases may also arise in these studies when GPs are responsible for patient inclusion even when they are supposed to apply rigorous inclusion criteria. Case-vignette studies offer an interesting alternate as they are easily implemented and accepted, avoid the selection biases mentioned above, and can control for specific patient characteristics [6], [15].

France is among the European countries with the highest rates of psychotropic drug use and the lowest rates of referral to specialists, despite the high per capita density of psychiatrists and the high level of other mental health resources [1], [16]. Continued medical education has been mandatory for French physicians since 1996. Good practice guidelines for the management of MD were issued by an independent agency in 2002 and have not been revised since. France's health care insurance funds currently have no provisions for performance payments as incentive to encourage GPs to reduce inappropriate antidepressant prescriptions.

Using a national panel of 1431 GPs in private practice that was set up in June 2010 to study GPs' attitudes and practices regarding different public health problems [17], we conducted a cross-sectional survey to study how GPs manage the acute phase of new cases of non-comorbid major depression (MD). The first specific aim was to describe GPs' choices among several treatment modalities: watchful waiting (assessment with scheduled follow-up but no active medication or psychotherapy treatment) [18]; initiating pharmacological treatment; recommending psychotherapy; providing psychological support. The second specific aim was to investigate the factors associated with these choices: we analyzed GPs' characteristics and attitudes toward different treatments for depression, MD severity [15] and patients' characteristics – gender and socioeconomic status (SES). We focused on these two patient characteristics, as it has been shown that GPs manage depression differently according to patient gender [8], [15], [19] and SES [8], [20]; we also analyzed the density of mental health specialists in each of the GPs' practice areas, as the supply of specialists may influence GPs' choices in managing MD [21]. The study aims were achieved.

## Materials and Methods

### Sampling

In 2008, around 58 000 GPs (31.6% of them women) were in private practice in France [22]. The survey described was the third of a series nested in the national panel of French GPs, designed to collect data regularly about their activity and practices. Its methodology has been presented elsewhere [17]. Briefly, 5170 GPs were randomly selected from the Ministry of Health's exhaustive database of health professionals in France. Sampling was stratified for location of the general practice (urban, suburban, or rural), gender, age (<49, 49–56, >56), and annual workload, defined by number of office consultations and house calls (<2849, 2849–5494, >5494) in 2008. Information on each GP was obtained from the General Health Insurance Fund. To limit a selection bias that might have resulted from particular opinions/attitudes, the specific topics to be studied were not mentioned to GPs before they were asked to participate in the panel.

Of the GPs contacted and eligible, 1431 (36.8%) agreed to join, i.e., to respond to 5 consecutive surveys during a 30-month period. The GPs who refused to take part did not differ from participants according to practice location, but were more frequently male (p = 0.02), older (p<10^−3^), and had a higher workload in 2008 (p<10^−3^). Lack of time (46.2%) and lack of interest in the panel (15.6%) were the reasons given most frequently for refusal [17].

### Ethics statement

GPs who agreed to participate in the panel sent back a signed written consent to our team. The National Data Protection Authority (Commission Nationale Informatique et Libertés), responsible for ethical issues and protection of computerized individual data in France, approved the panel and its procedures.

### Procedure and questionnaire

This cross-sectional survey took place during the last trimester of 2011. Professional investigators contacted the panel members and interviewed them with computer-assisted telephone interview (CATI) software. We developed a standardized questionnaire based on a previous qualitative study of the practices and attitudes of 32 GPs concerning the management of depression [23], a literature review, and discussions with experts; we pilot-tested it for clarity, length, and face validity among 50 GPs.

The questionnaire included case-vignettes — a method already been employed by several authors [6], [15] — about patients consulting for the first time about sadness, sleep problems, and loss of appetite over the previous three weeks and diagnosed with MD. These case-vignettes were designed in several versions that varied only according to patient gender and SES (blue collar/white collar) and MD severity (mild/severe). These 2×2×2 conditions produced 8 versions (see Appendix S1), and GPs were randomized to receive one of them [15]. Participants were first asked if they would manage these cases or refer the patient to a mental health specialist (referred to hereafter as “specialist”) for complete management (pharmacological and psychotherapeutic treatment). GPs who would continue to manage the cases were asked questions about the management they would choose: watchful waiting; provision of psychological support; prescription of pharmacological treatment and its type (antidepressant/anxiolytic/hypnotic); or referral for psychotherapy. The questionnaire also covered the GPs' professional (Table 1) and personal characteristics, their opinions about psychotherapy (efficacy, barriers to access), their cooperation with mental health specialists (utility, satisfaction), and their opinions and practices in the area of depression management (Table 2).

**Table 1:** Demographic and professional characteristics of GPs who participated and who did not participate in the survey† (French nationwide panel of general practitioners, weighted data, N = 1,249).

	Participants N = 1249 (%)	Non-participants N = 182 (%)
**Age (years)**		
<49	32.3	30.7
49–56	35.1	29.5
>56	32.6	39.8
**Gender**		
Male	72.9	70.6
Female	27.1	29.4
**Place of practice**		
Rural area	20.9	20.0
Suburban	18.6	15.2
Urban	60.5	64.8
**Number of visits and house calls in 2008**		
<2849	20.6	25.7
2849–5494	54.2	50.2
>5494	25.2	24.2
**Type of practice**		
Group	54.8	48.2
Solo	45.2	51.8
**Occasional practice of alternative medicine** ‡		
Yes	13.2	15.5
No	86.8	84.5
**Continuing medical education on depression**		
Never	23.2	NA
More than 3 years ago	49.6	
Less than 3 years ago	27.2	
**Participation in a mental health care network** §		
Yes	4.3	NA
No	95.7	

† no significant differences were found (at p = 0.05).

‡ homeopathy, acupuncture.

§ network of GPs and mental health specialists funded by the Regional Health Agency (Agence Régionale de Santé) and aimed at promoting access to and coordination of care.

**Table 2:** GPs' personal characteristics and attitudes about the management of depression (French nationwide panel of general practitioners, weighted data, N = 1249).

GPs characteristics	%
**Personal characteristics**	**Yes**
Personal history of depression (lifetime)	17.2
Personal history of consultation with a mental health specialist (lifetime)	15.5
Personal history of psychotherapy including psychoanalytic therapy (lifetime)	13.1
Personal history of antidepressant treatment (lifetime)	1.6/9.5/87.9/1.0†
History of depression in someone close to GP	42.2
**GPs' opinions about depression management**	**Agreed** ‡
Do you think that antidepressants are overprescribed in France?	44.8
Do you feel effective in managing patients with MD?	84.1
Do you feel that you are sufficiently trained to diagnose and treat patients with MD?	89.5
**GPs' practices in depression management**	**Often/very often**
What treatment do you usually propose to patients with depression of mild severity?	
A pharmacological treatment alone initially?	36.7
Psychotherapy alone initially?	27.6
**GPs' opinions about the effectiveness of psychotherapy**	**Agreed** ‡
“Psychotherapy constitutes treatments for MD just as antidepressants do”*	72.2
“Psychotherapy alone is effective in treating an episode of MD of mild-to-moderate severity”*	71.2
“In mild-to-moderate severe MD, the risk/benefit ratio is more favorable for psychotherapy than for antidepressants”*	61.3
“Psychotherapy is better suited for highly educated patients than for others”	59.2
**GPs' opinions about access to psychotherapy**	**Agreed** ‡
In your opinion, what are the barriers to referral for psychotherapy?	
“Consultations with psychologists and psychotherapists are not reimbursed”	90.9
“Delays for appointments with psychiatrists are too long”	78.6
“GPs have difficulties identifying the various kinds of psychotherapy”	65.8
“Patients are hesitant to get involved in psychotherapy”	76.3
**GPs' attitudes about mental health professionals**	**Agreed** ‡
Cooperation with mental health specialists:	
- Improves access to care**	73.9
- Improves care**	82.4
- Improves your skills**	69.0
“Are you satisfied with the cooperation with the mental health specialists to whom you refer your patients with depression?”	35.1§

† in the preceding year; more than 1 year ago; never; did not answer.

‡ agreed/totally agreed.

§ satisfied, very satisfied.

* Items entered in the calculation of the score of perception of the effectiveness of psychotherapy.

** Items entered in the calculation of the score of perception of the utility of cooperation with mental health specialists.

Finally, we obtained from the Ministry of Health data about the number of private-practice psychiatrists per 100 000 inhabitants in each GP's local area (specifically, “bassin de vie”, defined by the National Institute for Statistics and Economic Studies (INSEE) as the smallest area in which residents have access to usual daily services and work). GPs generally tend to refer their patients to psychiatrists in private practice, as public-sector psychiatrists primarily treat patients with severe mental illness.

### Statistical analysis

Because of the differences mentioned above between GPs who participated in the panel and those who refused, we weighted the data to match the sample more closely to the national French GP population for age, gender, location of practice, and 2008 workload [17], [24]. To take the sample stratification and weighting into account, we used Rao-Scott Chi-2 tests to examine bivariate associations.

We built a score of GPs' perceptions of the effectiveness of psychotherapy by summing the responses to 3 questions about these perceptions (alpha Cronbach = 0.70, Table 2) and a score of GPs' perceptions of the utility of cooperation with mental health specialists by summing the responses to 3 questions about these perceptions (alpha Cronbach = 0.84, Table 2).

We conducted weighted univariate and then multivariate logistic regressions of the factors associated with the following dependent variables: 1) among GPs who reported that they would start medical treatment by recommending antidepressants (versus any treatment not including antidepressants); 2) among GPs proposing antidepressants, recommending that they be combined with psychotherapy (versus without psychotherapy). All models were adjusted for GPs' gender, age, GPs' 2008 workload, and general practice location. Explanatory variables significant at p<0.15 in univariate analyses were introduced into the multivariate model and kept according to a backward selection procedure (exit criterion: p≥0.05). Model fit was assessed with the Hosmer-Lemeshow goodness-of-fit test. Analyses were performed with SAS version 9.2 (SAS Institute, Cary, NC).

## Results

Of the 1431 GPs initially included in the panel, 1249 (87.3%) agreed to participate in this survey; those who refused did not differ significantly from participants according to gender, age, place of practice or number of office visits and house calls in 2008 (Table 1). Table 2 reports GPs' personal characteristics and opinions about treatment for major depression. Most GPs felt they were adequately trained to treat major depression and that they did so effectively. A majority of GPs had a favorable perception of the effectiveness of psychotherapy but only 35% found that cooperation with mental health specialists was satisfactory (Table 2).

**Figure 1:**
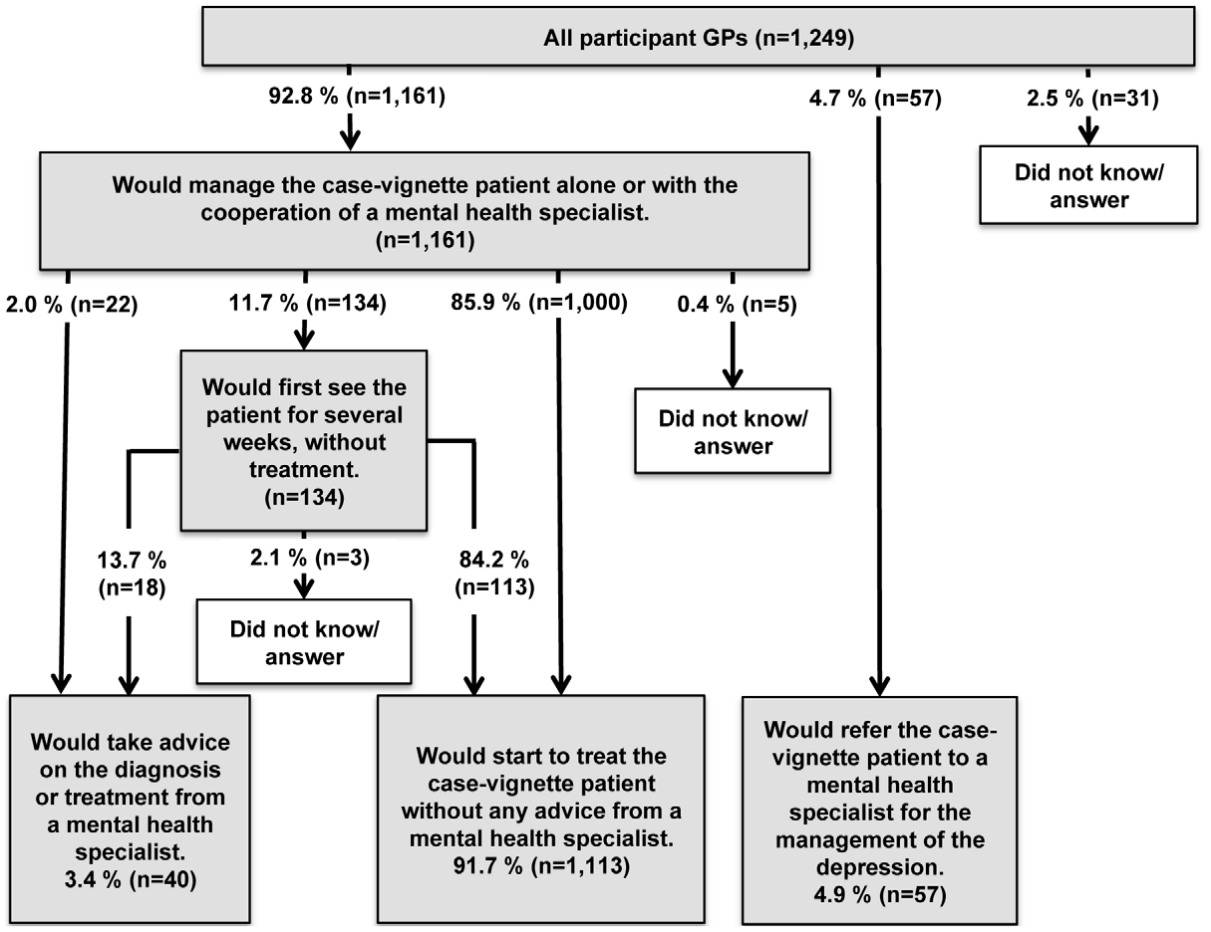
Initial management of the case-vignette patients with major depression by GPs (Weighted data, N = 1,249).

**Figure 2:**
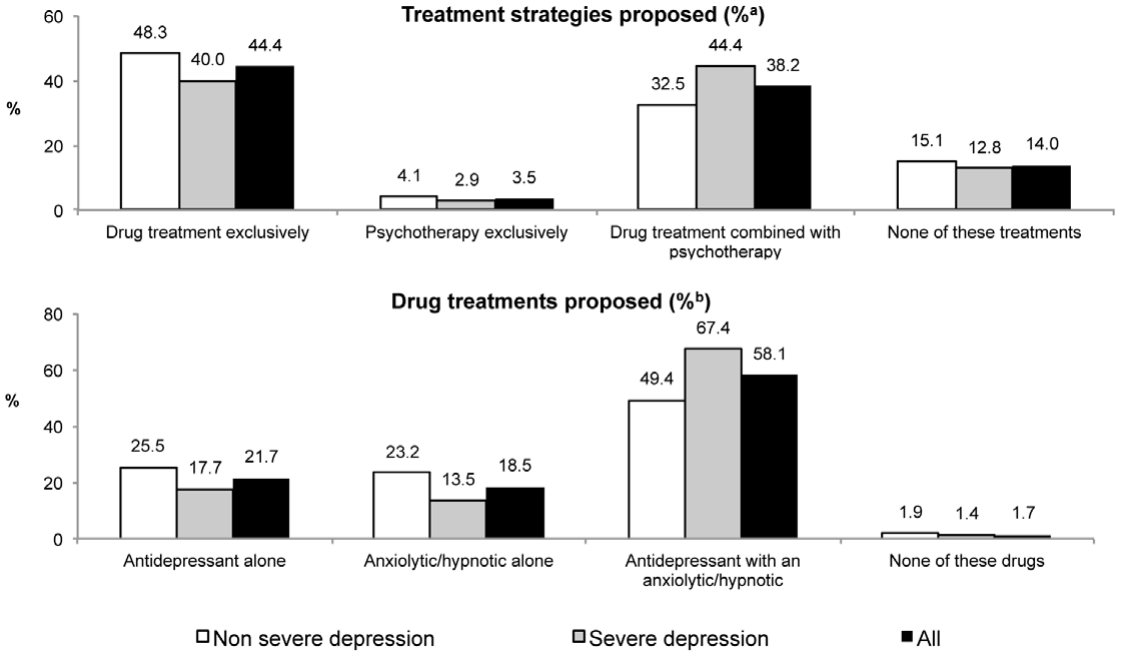
GPs' treatment choices according to major depression severity (Weighted data). ^a^ Calculated among GPs managing the patient themselves without any specialist's advice and GPs who would take advice on the diagnosis before managing the patient ^b^ Calculated among GPs recommending a drug treatment.

The great majority of GPs would manage cases of MD themselves, without referring the patient directly to a mental health specialist, although some would seek advice from a specialist. Figure 1 presents the GPs' initial choices for all of the case-vignettes. Note that these choices varied according to severity: 84.7% of GPs assigned a mild case-vignette would choose to start active treatment immediately without a specialist's advice, while 13.9% said that they would opt for watchful waiting; in the severe cases the corresponding figures were 87.2% and 9.3% (p<0.01). Sixty percent of GPs would provide psychological support in mild cases and 68.4% in severe ones (p<0.01; total percentage: 64.0%). The percentages of GPs who chose exclusive pharmacotherapy, pharmacotherapy combined with psychotherapy, and psychotherapy alone are presented in Figure 2. These too differed significantly according to severity (p<0.01). Differences according to severity were also found among the participants who chose pharmacotherapy regarding the kinds of psychotropic drugs proposed (p<0.001, Figure 2): 74.9% who were randomized to case-vignettes of mild MD would choose an antidepressant and 85.1% of those responding to a severe case (p<0.001). Among GPs who reported that they would manage the cases (whatever their treatment choice) the total percentage of GPs choosing antidepressants was 66.2%. Among GPs who reported that they would suggest antidepressant treatment, 41.4% also said they would propose psychotherapy in mild cases and 54.0% in severe cases (p<0.001; total percentage: 47.9%).

In the first multivariate logistic regression (Table 3), the choice of antidepressants was negatively associated with the doctor being a woman, having had psychotherapy, and believing that antidepressants are over-prescribed in France, and with the patient being a woman. It was also positively associated with severity, the GP's personal or family history of depression, and the GP's personal history of antidepressant treatment. GPs who generally often/very often treated mild to moderate MD with pharmacotherapy alone also chose antidepressant treatment more often than the other GPs. Neither the professional characteristics of GPs nor the local density of private psychiatrists were associated with this first dependent variable.

**Table 3:** Factors associated with the prescription of antidepressant (ATD) treatment versus no antidepressant (Case-vignette, French nationwide panel of general practitioners, weighted multivariate logistic model, N = 1097).

		GPs' treatment choices		
		Not an ATD (%)	ATD (%)	Odds ratio [95% confidence interval]
**All**		**33.8**	**66.2**	
**GPs' personal characteristics**				
Age† (years)	<49	38.8	28.8	__
	49–56	31.2	37.8	1.39 [1.00;1.92]
	>56	30.0	33.4	1.15 [0.82;1.61]
Gender†	Male	67.2	76.9	__
	Female	32.8	23.1	0.69 [0.51;0.95]
History of depression in someone close	No	60.8	55.9	__
	Yes	39.2	44.1	1.34 [1.02;1.76]
Personal history of psychotherapy	No	83.6	88.3	__
	Yes	16.4	11.7	0.57 [0.37;0.87]
Personal history of antidepressant treatment	No	92.4	86.4	__
	Yes	7.6	13.6	2.31 [1.41;3.81]
**GPs' opinion about depression treatments**				
In depression of mild/moderate severity, starts treatment with a drug therapy only	Never	26.0	14.8	__
	Sometimes	43.1	45.3	1.75 [1.23;2.48]
	Often/very often	30.9	39.9	2.05 [1.42;2.97]
Thinks that antidepressant are over-prescribed in France	Totally disagrees/partially disagrees	47.8	60.6	__
	Partially agrees/totally agrees	52.2	39.4	0.63 [0.48;0.82]
**Patient's personal characteristics and depression severity**				
Intensity of depression	Mild	60.2	48.5	__
	Severe	39.8	51.5	1.74 [1.33;2.27]
Patient's gender	Male	44.2	51.9	__
	Female	55.8	48.1	0.75 [0.58;0.98]

† Sample stratification variables were forced in the model even if they did not met the p<0.05 criterion; among them, the number of visits and house calls in 2008 and the place of practice were not significantly associated to the dependent variable (results not presented).

Hosmer-Lemeshow test: p = 0.51. 25 GPs excluded because of missing values.

The following variables were not selected after the univariate regressions (p>0.15): type of practice, occasional practice of alternative medicine, personal history of consultation of a mental health specialist, score of perception of the utility of and satisfaction about GP-mental health specialist cooperation, GPs' opinions regarding their training and effectiveness in depression management and patient's SES, density of private-practice psychiatrists in each GPs' local area.

The following variables were rejected by the backward process in the final multivariate model: CME on depression diagnosis and management, personal history of depression, participation in a formal mental health care network, and GPs' opinions regarding barriers to access to psychotherapy in their practice area.

In the second multivariate logistic regression (Table 4), a combination of antidepressants and psychotherapy was negatively associated with GPs' age, their opinion that they are adequately trained to manage depression, and a low level of satisfaction about cooperation with mental health specialists. Choosing this combination was positively associated with the doctor being a woman, an intermediate or high score for favorable perception of the effectiveness of psychotherapy in treating depression, a high score for perception of the utility of cooperation with mental health specialists, severity of depression, and high patient SES. The GPs' professional characteristics, their opinions on barriers to access to psychotherapy, and private psychiatrists density were not associated with this second dependent variable.

**Table 4:** Factors associated with the prescription of antidepressant treatment combined with psychotherapy versus an antidepressant alone (versus antidepressant therapy only, case-vignette, French nationwide panel of general practitioners, weighted multivariate logistic model, N = 720).

		GPs' treatment choices		
		ATD without psycho-therapy (%)	ATD with psycho-therapy (%)	Odds ratio [95% confidence interval]
**All**		**33.8**	**66.2**	
**GPs' personal characteristics**				
Age†	<49	22.1	36.2	__
	49–56	37.7	37.2	0.67 [0.45;1.00]
	>56	40.2	26.6	0.49 [0.32;0.76]
Gender†	Male	83.3	69.8	__
	Female	16.7	30.2	1.76 [1.17;2.63]
**GPs' opinion about depression treatments**				
Thinks that she/he is sufficiently trained to manage depression	Totally disagrees/partially disagrees	10.3	18.9	__
	Partially agrees/totally agrees	89.7	81.1	0.57 [0.36;0.91]
Score of perception of the effectiveness of psychotherapy in treating depression‡	Low (0–6)	38.4	22.7	__
	Intermediate (7–9)	44.7	53.2	1.88 [1.29;2.73]
	High (10–12)	16.9	24.1	1.73 [1.09;2.77]
**GPs' opinion on cooperation with mental health specialists**				
Satisfaction about the cooperation with mental health specialists	Yes	29.3	40.6	__
	No	70.7	59.4	0.63 [0.45;0.89]
Score of perception of the utility of cooperation with mental health specialists§	Low (0–8)	43.1	28.0	
	Intermediate (9–10)	36.1	29.7	1.28 [0.87;1.88]
	High (11–12)	20.8	42.3	3.08 [2.06;4.62]
**Patient's personal characteristics and depression severity**				
Intensity of depression	Mild	54.6	41.8	__
	Severe	45.4	58.2	1.82 [1.31;2.52]
Patient's SES	Blue-collar	54.8	43.1	__
	White-collar	45.2	56.9	1.58 [1.14;2.18]

† Sample stratification variables were forced in the model even if they did not meet the p<0.05 criterion; among them, the number of visits and house calls in 2008 and the place of practice were not significantly associated to the dependent variable (results not presented).

‡ Score transformed into a three categorical variable according to its first and third tertiles values (min = 0; Q1 = 6; Q3 = 9; max = 12).

§ Score transformed into a three categorical variable according to its first and third tertiles values (min = 0; Q1 = 6; Q3 = 11; max = 12).

Hosmer-Lemeshow test: p = 0.60. 8 GPs excluded because of missing values.

The following variables were rejected during the univariate selection process (p>0.15): type of practice, occasional practice of alternative medicine, personal history of depression/psychotherapy/antidepressant treatment and participation in a formal mental health care network, density of private-practice psychiatrists in each GPs' local area.

The following variables were rejected by the backward process in the final multivariate model: CME on depression diagnosis and management, family history of depression, personal history of consultation of a mental health specialist, thinks that antidepressant are over-prescribed in France, and GPs' opinions regarding barriers to access to psychotherapy in their practice area.

## Discussion

This study is to our knowledge the first national study to use a randomized case-vignette methodology to conduct a global assessment of how primary care physicians manage patients with newly diagnosed non-comorbid MD. Among GPs responding to these case-vignettes, nearly two-thirds would provide psychological support, and the great majority would propose pharmacotherapy, with or without psychotherapy. Among the latter, 79.8% would choose an antidepressant (with or without anxiolytic/hypnotic) and 18.5% an anxiolytic/hypnotic alone. Respondents rarely chose psychotherapy alone, regardless of case severity. Antidepressant prescription was associated with severity, patient gender, and GP personal characteristics. The combination of antidepressant treatment and psychotherapy was associated with severity of depression, patient SES, and GPs' opinions, especially about the quality of cooperation with mental health specialists in treating depression.

### Active treatment versus watchful waiting

As observed in previous case-vignette studies [6], [18], GPs were much more likely to suggest immediate (85.9%) active treatment than watchful waiting (11.7%). Moreover the percentage for the latter, substantially lower than in earlier studies (20%–41%), suggests that French GPs are mostly inclined to start active treatment for MD immediately, regardless of its severity. Reasons may include their sense of effectiveness in managing this disease and their dissatisfaction about access to and cooperation by mental health specialists (Table 2). This conduct is in line with French guidelines and those in other countries (e.g. the CANMAT guidelines in Canada), given that an MD diagnosis *per se* implies the need for immediate treatment [10], [25]. However, the NICE clinical guidelines (UK) allow “active monitoring” for patients who, in the practitioner's judgment, may recover without any formal intervention, or have mild depression and do not want intervention, or have sub-threshold depressive symptoms and request an intervention [2]. Some authors suggest that watchful waiting might reflect the GP's uncertainty about the diagnosis and treatment utility [26]. In the French context, where most GPs have confidence in their ability to manage MD, this choice might also reflect disagreement with the guidelines.

### Psychological support by GPs

A relatively high percentage of GPs in our study would provide psychological support to patients with MD. This is in line with previous publications showing that family physicians attach much importance to giving such support to depressed patients [27], [28]. It is also something patients expect [29]. The omission of any mention of this in French guidelines for depression management [10] suggests, however, that GPs have constructed their own vision of how to manage MD.

### Pharmacotherapy

The percentage of French GPs choosing pharmacotherapy first (with or without psychotherapy, 82.6%) was in the upper range of the figures published in other studies of family physicians (34%–94%) reviewed by Kosteniuk et al. [6]. Among GPs recommending pharmacotherapy, a non-negligible percentage (18.5%) recommended anxiolytic/hypnotic drugs alone (Table 2). GPs are likely to prescribe anxiolytics or hypnotics to patients with major depression [14], [16], [30], [31] even though these drugs are not recommended alone to treat this disease, even when its intensity is mild [10], [32], [33]. Guidelines in France [10] recommend prescribing these drugs when starting an antidepressant treatment for the shortest possible period only in case of marked symptoms of anxiety, agitation or insomnia, symptoms not present in our case-vignettes.

### Antidepressant treatment and associated factors

Although antidepressants remained the treatment chosen most frequently by our study's participants (66.2%), this percentage is moderate compared with other studies, where it sometimes exceeded 80% [13], [34]–[36]. Our results suggest that some GPs hesitate to prescribe antidepressants even in severe MD. Indeed, GPs do not unanimously believe that antidepressants universally produce satisfactory results [5], [15]. They may also anticipate that patients will be reluctant to use antidepressants and plan several consultations to overcome this reluctance [37]. Finally, some GPs may have internalized the French public authorities' messages about the over-prescription of psychotropic drugs (Table 2), as suggested by the slight decrease in antidepressants prescribed in France in 2006–2009 (minus 1% per year) [38]. These messages must nonetheless be carefully tailored to avoid dissuading GPs from prescribing antidepressants when these drugs are indicated.

GPs' personal life experiences of depression, psychotherapy, and antidepressant treatment influenced their choice to use antidepressants when professional characteristics in particular CME did not (Table 3). Swedish studies [37], [39] found that GPs consider their personal qualities and life experiences more influential in treating depression than academic education and professional literature. This may lead them to make non-optimal choices in managing MD. GPs' education on this topic should incorporate the results of studies such as these to increase awareness of the possible gap between their practices and recommendations and evidence-based medicine that may occur when relying more on life experience rather than on scientific evidence.

Our finding that GPs would prescribe antidepressants less often to women than men is consistent with some publications [8], but contradicts others, which either found no association [13], [40] or an inverse one [41], [42]. Gender differences in diagnosis and treatment of depression found in population studies [16] have been ascribed to women seeking medical care more often than men, verbalizing their symptoms more effectively, and requesting medication more often [15]. There are several possible reasons for the higher level of antidepressant prescriptions in our male vignettes, however: depressive symptoms of similar intensity may be perceived by GPs as more severe in men, and GPs may take into account the higher risk of suicide in men than in women [8].

### Psychotherapy and associated factors

The percentage of GPs who would recommend pharmacotherapy alone and in combination with psychotherapy was similar, as previously observed in case-vignettes [6] and population studies [16]. In all, about 40% of GPs recommended referral for psychotherapy, which is strikingly close to the rate of psychotherapy among patients with mood disorders (47%) in the ESEMeD population study [16]. Nonetheless, GPs were reluctant to propose psychotherapy alone as a first-line treatment (3.5%) even in mild MD, when it is especially recommended by, among others, French, British, and US guidelines [2], [10], [32]. In other studies reviewed by Kosteniuk et al. [6], 7% to 47% of family physicians would recommend psychotherapy to treat MD. Despite relatively favorable perceptions of the effectiveness of psychotherapy in treating MD (2/3 of the GPs in our study had an intermediate-to-high score for this perception, Table 3), the majority recommended psychotherapy only as a complement to antidepressants, as reported elsewhere [39].

GPs chose the combination of antidepressant drugs and psychotherapy more frequently in the severe than the mild cases (Table 4). This is consistent with French and international guidelines [10], [32], although the latter also recommend this combination in mild to moderately severe MD when specific conditions are present (e.g., psychosocial, interpersonal problems, or an axis II disorder). Low patient SES appeared to be a barrier to this combination (Table 4). A previous study in France found that psychotherapy is underutilized by depressed patients with a low educational level [43]. Psychotherapy in France is, at best, only partially reimbursed, and frequently not at all; this policy choice might partially explain our results, as might the attitude of many GPs, who considered psychotherapy better suited to highly educated patients than to others (Table 2).

GPs' perceptions of the utility of cooperation with mental health specialists and their satisfaction with it, their perceptions of both the effectiveness of psychotherapy and the adequacy of their own training were the main attitudinal factors associated with their decision to combine antidepressants and psychotherapy (Table 4). On the other hand, neither their opinions about the barriers related to the provision of and/or access to psychotherapy nor the density of private psychiatrists in their practice areas were associated with this recommendation. A previous population-based study in France did, however, find this density to be associated with lifetime recourse to psychotherapy [43]. Environmental constraints regarding access to psychotherapy might have a substantially greater impact on patient uptake than on their GPs' recommendations.

### Limits and strengths of the study

The participation rate in this survey was high, reflecting good adherence to the panel. Moreover, GPs who did not participate in this survey did not differ from participants for gender, age, place of practice, or 2008 workload. The survey was cross-sectional: therefore, the observed links should be interpreted with caution. Nonetheless, the quasi-experimental design we followed in applying the case-vignette strengthens our findings especially as to patient gender and SES and disease intensity, because it allowed us to control these characteristics [44]. Moreover, the vignette method reduced bias by presenting patients who were standardized [45] and allowed us to include almost all panel participants [15]. Although the design and wording of the case-vignette was as close as possible to a real situation, this method cannot ensure that responses reflect the way GPs would behave in a real situation [14]: it cannot take into account the doctor-patient interactions that shape clinician behavior [6]. However it had acceptable face validity with different degrees of MD severity leading to different management [15], and its results were consistent with those of a population study [16].

### Conclusions

In this case-vignette study of new cases of MD, GPs made decisions that complied better with clinical guidelines for severe cases than for mild ones. Although most GPs were favorable to psychotherapy, their choices indicate that they did not use it as stand-alone treatment for depression. At the same time, some of them appeared careful in recommending antidepressants when starting MD treatment, which suggests that they have internalized the messages of French public health authorities about over-prescription of antidepressants in France. Finally, GPs' decisions about the treatment choices were influenced mainly by their personal characteristics and life experience, their attitudes toward treatments and patients' gender and SES, but not by continuing medical education or barriers to access to psychotherapy and/or specialists. These results raise questions about the methods, models, and content of continuing medical education used in France for the management of MD. Developing new educational programs to increase GPs' knowledge of the different treatment strategies for MD and their indications and improving cooperation between them and mental health specialists are necessary steps for improving access to adequate treatment for primary care patients.

## Supporting Information

Appendix S1 (DOC)
