# Supplementary material for: General Practitioners' Choices and Their Determinants When Starting Treatment for Major Depression: A Cross Sectional, Randomized Case-Vignette Survey
Source: PLoS One. 2012 Dec 18;7(12):e52429. doi: 10.1371/journal.pone.0052429 (PMC3525552; doi:10.1371/journal.pone.0052429)
Supplement: Appendix S1 — (DOC) [file pone.0052429.s001.doc]

**Appendix 1**

1. A 40-year-old **man**, a **blue-collar worker**, comes to see you because for more than 3 weeks he has been experiencing, for the first time, continued sadness, sleep problems, and loss of appetite. Your examination indicates he is depressed and that its functional effect on his daily life is **severe: he is not able to work**. He takes no medications, reports low alcohol consumption, and you find no physical or traumatic cause, or any recent event in his work or personal life that might explain this depression. No suicidal ideation or other mental or physical illness is present.

2. A 40-year-old **man**, a **blue-collar worker**, comes to see you because for more than 3 weeks he has been experiencing, for the first time, continued sadness, sleep problems, and loss of appetite. Your examination indicates he is depressed and that its functional effect on his daily life is **mild: he is able to work**. He takes no medications, reports low alcohol consumption, and you find no physical or traumatic cause, or any recent event in his work or personal life that might explain this depression. No suicidal ideation or other mental or physical illness is present.

3. A 40-year-old **man**, a **manager**, comes to see you because for more than 3 weeks he has been experiencing, for the first time, continued sadness, sleep problems, and loss of appetite. Your examination indicates he is depressed and that its functional effect on his daily life is **severe: he is not able to work**. He takes no medications, reports low alcohol consumption, and you find no physical or traumatic cause, or any recent event in his work or personal life that might explain this depression. No suicidal ideation or other mental or physical illness is present.

4. A 40-year-old **man**, a **manager**, comes to see you because for more than 3 weeks he has been experiencing, for the first time, continued sadness, sleep problems, and loss of appetite. Your examination indicates he is depressed and that its functional effect on his daily life is **mild: he is able to work**. He takes no medications, reports low alcohol consumption, and you find no physical or traumatic cause, or any recent event in his work or personal life that might explain this depression. No suicidal ideation or other mental or physical illness is present.

5. A 40-year-old **woman**, a **blue-collar worker**, comes to see you because for more than 3 weeks she has been experiencing, for the first time, continued sadness, sleep problems, and loss of appetite. Your examination indicates she is depressed and that its functional effect on her daily life is **severe**: **she is not able to work**. She takes no medications, reports low alcohol consumption, and you find no physical or traumatic cause, or any recent event in her work or personal life that might explain this depression. No suicidal ideation or other mental or physical illness is present.

6. A 40-year-old **woman**, a **blue-collar worker**, comes to see you because for more than 3 weeks she has been experiencing, for the first time, continued sadness, sleep problems, and loss of appetite. Your examination indicates she is depressed and that its functional effect on her daily life is **mild: she is able to work**. She takes no medications, reports low alcohol consumption, and you find no physical or traumatic cause, or any recent event in her work or personal life that might explain this depression. No suicidal ideation or other mental or physical illness is present.

7. A 40-year-old **woman**, a **manager**, comes to see you because for more than 3 weeks she has been experiencing, for the first time, continued sadness, sleep problems, and loss of appetite. Your examination indicates she is depressed and that its functional effect on her daily life is **severe: she is not able to work**. She takes no medications, reports low alcohol consumption, and you find no physical or traumatic cause, or any recent event in her work or personal life that might explain this depression. No suicidal ideation or other mental or physical illness is present.

8. A 40-year-old **woman**, a **manager**, comes to see you because for more than 3 weeks she has been experiencing, for the first time, continued sadness, sleep problems, and loss of appetite. Your examination indicates she is depressed and that its functional effect on her daily life is **mild: she is able to work**. She takes no medications, reports low alcohol consumption, and you find no physical or traumatic cause, or any recent event in her work or personal life that might explain this depression. No suicidal ideation or other mental or physical illness is present.
